# Skin autofluorescence is associated with blood glucose levels, especially in children with type 1 diabetes

**DOI:** 10.3389/fcdhc.2025.1590288

**Published:** 2025-06-30

**Authors:** Tinghan Deng, Jingping Wu, Hongbin Cheng

**Affiliations:** ^1^ Clinical Research on Skin Diseases, School of Clinical Medicine, Chengdu University of Traditional Chinese Medicine (TCM), Chengdu, Sichuan, China; ^2^ Department of Medical Cosmetology, Hospital of Chengdu University of Traditional Chinese Medicine, Chengdu, Sichuan, China; ^3^ Dermatology of Department, Hospital of Chengdu University of Traditional Chinese Medicine, Chengdu, Sichuan, China

**Keywords:** skin autofluorescence, diabetes mellitus, children, diagnosis, meta-analysis

## Abstract

**Background:**

This study examines the correlation between skin autofluorescence (SAF) and blood glucose levels, emphasizing the accumulation of advanced glycation end-products (AGEs). We hypothesize that SAF levels are closely linked to type 1 diabetes complications in children. The aim is to evaluate SAF’s relationship with type 1 diabetes progression in children and its potential as a non-invasive tool for disease detection and monitoring complications. The research was registered with PROSPERO (CRD42021284774).

**Methods:**

We conducted a meta-analysis by extracting studies from databases including PubMed, MEDLINE, EMBASE, Cochrane, Science Direct, Scopus, and Web of Science. A random effects model was used to assess if SAF measurement could serve as a non-invasive marker for type 1 diabetes and its complications. SAF values were compared between children with type 1 diabetes and controls, calculating the mean difference and 95% confidence intervals.

**Results:**

The analysis included three case-control studies and one retrospective cohort study, all using the AGE Reader^®^ (DiagnOptics Technologies). Data analysis showed significant heterogeneity (I² = 82%, P < 0.05). The random effects model revealed a positive correlation between higher SAF levels and type 1 diabetes in children [mean difference = 0.20 (0.16, 0.25)], indicating elevated SAF in diabetic children compared to non-diabetic peers.

**Conclusion:**

This research supports SAF measurement as a non-invasive indicator for type 1 diabetes and its complications in children. However, further studies with larger samples and longer follow-up are needed for definitive conclusions and detailed insights into complications. Additionally, the skin’s multifaceted roles require further investigation.

**Systematic Review Registration:**

https://www.crd.york.ac.uk/prospero/, identifier CRD42021284774.

## 1 Introduction

Type 1 diabetes (T1D) is a diverse condition marked by the destruction of pancreatic β cells, resulting in a complete lack of insulin. Most T1D patients experience β-cell destruction due to autoimmune responses (type 1), while others suffer from a distinct form of β-cell destruction or failure (type 1b). T1D represents 5%–10% of all diabetes cases worldwide, with an increasing incidence rate of about 2%–3% annually (1, 2). Although less prevalent, T1D represents the predominant form of diabetes in children and adolescents, markedly affecting their healthy growth. The incidence and severity of diabetes, along with its complications, are higher in youth compared to adults. Research is crucial in preventing the onset of diabetes and its complications (3–5).

Before our present understanding of T1D, there were various theories and reports about its genetic and epidemiological aspects, immune responses, β-cell functionality, and overall impact. Diagnosis typically involves detecting fasting blood glucose levels over 7.0 mmol/L (126 mg/dl), with diabetes also diagnosable through a random blood glucose concentration exceeding 11.1 mmol/L (200 mg/dl) or an oral glucose tolerance test. Diagnosis without symptoms requires abnormal blood sugar readings on two separate occasions. Diabetes may also be diagnosed when hemoglobin A1c (HbA1c) levels exceed 48 mmol/mol (6.5%). However, due to the rapid fluctuation of blood glucose in T1D patients, HbA1c is not the most reliable diagnostic tool (3). HbA1c measurements are useful for monitoring short-term blood glucose control. The principle involves obtaining blood from red blood cells (lifespan approximately 100–130 days), where HbA1c forms through the irreversible glycation of hemoglobin. HbA1c levels reflect blood glucose control over the past 1–3 months but are influenced by various factors (6, 7). For children with type 1 diabetes, the frequent need to collect blood for glucose monitoring is burdensome. Thus, it becomes crucial to explore non-invasive approaches for long-term blood sugar management in these young patients. This study suggests that advanced glycation end-products (AGEs) can offer innovative ways to monitor blood glucose levels non-invasively.

The underlying theory is that as we age, long-lived proteins like skin collagen accumulate more rapidly in T1D patients. Skin collagen, with a half-life of 10–15 years, allows skin AGEs (sAGEs) to reflect extended blood sugar levels, adding value to patient monitoring over long periods (8, 9). Normally, the body eliminates AGEs and intermediates through enzymatic reactions. Macrophages and vascular endothelial cells in organs like the liver and lungs absorb and degrade AGEs through receptor-mediated responses. However, in diabetic patients, AGEs are produced faster than they can be eliminated, leading to their accumulation in the body. Changes in serum AGEs levels are crucial indicators of diabetes severity and its complications, closely linked to HbA1c and urinary albumin excretion rates. The accumulation of AGEs is also linked to potential future complications of diabetes (10). Skin autofluorescence (SAF) measurements offer a non-invasive method to assess the accumulation of sAGEs (11).

Researchers have developed innovative, rapid, and reliable non-invasive methods for measuring tissue levels of advanced glycation. Presently, AGEs in the human body are primarily detected using liquid chromatography-mass spectrometry (LC-MS) and enzyme-linked immunosorbent assay (ELISA). AGEs levels in human serum and skin homogenates, ascertained via tissue biopsy, are measured by these methods, while in skin, they are detected *in vitro* using a fluorescence detector. Comparative analysis reveals differences between quickly metabolized serum AGEs and those persisting in tissues; the latter being more stable and representative for evaluating AGEs accumulation in tissues. Initially, skin AGEs detection involved invasive methods, where biopsy tissues were analyzed for AGEs levels using LC-MS and ELISA. However, this method’s drawbacks—time-consuming, costly, with high infection risk and patient discomfort—make it unsuitable for clinical use. AGEs, appearing brownish-yellow and fluorescing upon excitation, owe these properties to the glycosides and pyrrolines within. Recently, fluorescence measurement for detecting skin AGEs levels has gained widespread adoption in clinical practice. Meerwaldt and colleagues assessed diabetes patients’ skin AGEs via autofluorescence and compared these levels to healthy subjects using skin biopsies. *In vitro* tests on skin tissue homogenates for AGEs and their fluorescence levels confirmed the viability of non-invasive skin AGEs detection (12, 13). Importantly, AGEs comprise various substances, some non-autofluorescent like carboxymethyllysine (CML). Research shows consistent levels of non-fluorescent AGEs in skin, whether detected non-invasively or via biopsy, confirming the reliability of non-invasive AGEs detection. The AGE Reader^®^ uses light in the 300–420 nm spectrum to illuminate a 2–4 cm area on the lower arm, with peak AGE fluorescence excitement at 370 nm, and a spectrometer measures the skin fluorescence across the 300–600 nm range.

Numerous studies indicate that skin autofluorescence (SAF) is valuable in diabetes screening and predicting complications, including microvascular and macrovascular diseases, and diabetic nephropathy. However, most of these studies focus on adults, overlooking the pediatric population (14–16). As dermatological researchers, our team prioritizes studies on skin-related conditions. Considering the skin’s status as the largest organ in the body, its diverse functions merit deeper exploration and research.” This revision simplifies the sentence while maintaining its original meaning, effectively reducing redundancy. This review and analysis represents the first study to use non-invasive tools for clinically verifying SAF in children with T1D. Particularly in predicting early complications, this method offers insights for the clinical management and control of T1D in children.

## 2 Method

As per Prisma guidelines for systematic reviews and analyses, our study, being data-based, is exempt from institutional review board approval.

### 2.1 Subsection

#### 2.1.1 Search strategy

Our comprehensive literature search encompassed multiple databases, including PubMed, MEDLINE, EMBASE, Cochrane Central Register of Controlled Trials, ScienceDirect, Scopus, and Web of Science, spanning publications from their inception until the search date. The clinical trials registry at clinicaltrials.gov was also reviewed for the period 2000-2021. Our strategy combined terms related to childhood diabetes and skin autofluorescence, using search phrases like “skin autofluorescence”, “skin fluorescence”, “Self-fluorescence of skin”, “SAF”, “Diabetes”, “Diabetes Mellitus”, “Diabetic patients”, “DM”, “children”, “child”, “kid”, and their variations with the Boolean operator “and”, without language restrictions (See **Table 1**). We extended our search by scrutinizing the reference lists of pertinent studies and reviews, thoroughly examining referenced reports’ full texts to find eligible studies, and contacting authors as needed for additional information.

**Table 1:** Strategy for identifying eligible studies in database searches.

“Diabetes” OR “Diabetes Mellitus” OR “Diabetic patients” OR “DM”	AND	“skin autofluorescence” OR “skin fluorescence” OR ‘Self-fluorescence of skin” OR “SAF”	AND	“children” OR “child” OR “kid”

### 2.2 Study inclusion

Criteria for Inclusion:

- Participants with Type 1 Diabetes (T1D), regardless of complications;
- Aged under 18;
- Any primary or secondary use of skin fluorescence for diabetes diagnosis.
- Exclusion criteria include:
- Studies without relevant findings or lacking association between skin autofluorescence (SAF) and diabetes mellitus (DM) characteristics or complications;
- Non-human research;
- Social commentaries, research proposals, or conference posters; studies not meeting inclusion criteria.

### 2.3 Study quality evaluation

Ms. Deng and Dr. Cheng independently reviewed the titles, abstracts, and full texts of retrieved literature, with Dr. Wu resolving any disagreements and making final decisions on exclusions. The two researchers then independently assessed the included studies using the Newcastle-Ottawa Scale, as outlined in **Table 2** (21). The quality of these studies was scored on this scale, categorizing them as low (0–3), moderate (4–6), or high quality (7–9), with 9 being the highest score. A black five-pointed star indicates that the assessment criterion meets the requirements and earns 1 point. A hollow five-pointed star indicates that the assessment criterion does not meet the requirements and a score of 0 is obtained. Blank items in the table indicate that the standard has not been evaluated, the data is missing or not applicable, and will not be included in the total score calculation.

**Table 2:** Assessment of study quality using the Newcastle-Ottawa Scale in meta-analysis.

Author, year	Selection				Comparability	Exposure			Scores
	Adequate definition of the case	Representativeness of the cases	Selection of controls	Definition of controls	Control for important factors	Ascertainment of exposure	Same method of ascertainment for cases and controls	Non-response rate	
Pascal, 2012 (17)	★	★	☆	★	★	☆	★	☆	7
Josine, 2016 (18)	★	★	☆	★	★	★	★	★	8
Y.H. Cho, 2014 (19)	☆	☆		★	☆		★	☆	4
Y.H. Cho, 2016 (20)	★	★	☆	★	★	★	★	☆	7

### 2.4 Data extraction

Two reviewers independently conducted data collection, resolving any discrepancies through discussion or consultation with a third reviewer. The review process involved a blinded, layered approach: initially screening titles and abstracts, then thoroughly reading the full texts, and finally selecting studies that met inclusion and exclusion criteria. Criteria for included literature encompassed author name, publication year, research institute location, study design, and participant characteristics such as sample size, average age, diabetes duration, hemoglobin A1c levels, and SAF values. Differences were resolved through discussion, and authors were contacted for any missing data.

### 2.5 Data synthesis and statistical analysis

Meta-analysis was conducted using Review Manager version 5.4 and Stata MP 14. A forest plot estimated the SAF value differences between children with T1D and healthy controls. The DerSimonian and Laird method analyzed the 95% confidence interval correlation coefficient. Cochrane’s Q statistic and the I² statistic assessed research heterogeneity, with high heterogeneity (I² ≥ 75%) triggering a sensitivity analysis to identify causes and confirm result stability. In cases of no heterogeneity (I² = 0), a fixed-effects model was applied, while a random-effects model was used for significant heterogeneity. Results with a p-value of ≤0.05 were considered statistically significant.

## 3 Results

### 3.1 Search results and characteristics of studies

**Figure 1:**
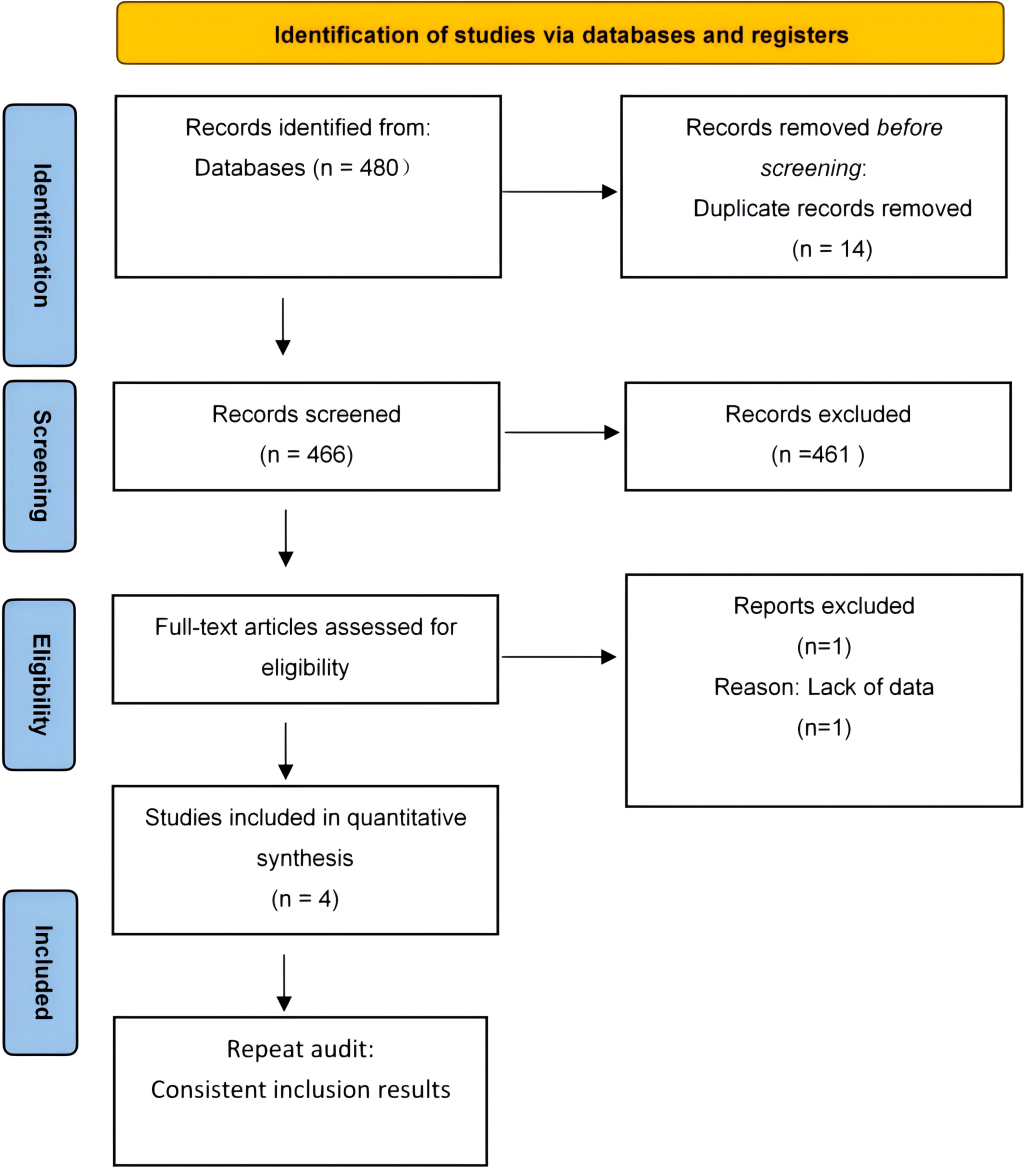
Flow diagram of included studies.

The initial database search yielded 480 records, including 14 duplicates. After removing these, 461 of the remaining 466 records were excluded for not meeting the criteria, resulting in five qualified studies. Subsequently, four studies were further excluded due to reasons such as insufficient data and unreachable authors, leaving only one study for inclusion. **Figure 1** illustrates this selection process in the PRISMA flowchart.

**Table 3** details the characteristics of the included studies, published between 2012 and 2016. These studies encompass three comparative case studies and one retrospective cohort study, conducted in France, the Netherlands, and Australia, with two in Australia. Each study compared a group of children with Type 1 Diabetes (T1D) to a control group of healthy children, totaling 342 children with T1D and 256 healthy children aged 6–18 years. Among the children with diabetes, 60.81% were boys; however, gender data was missing from one study due to an unreachable author. The duration of T1D in participants ranged from 2 to 14.1 years, with HbA1c levels between 7.16% and 10.2%. SAF values in all studies were measured using the AGE Reader^®^ (DiagnOptics Technologies, Groningen, The Netherlands), as described in **Table 3**.

**Table 3:** Features of selected studies.

Author, year	Study design	Location	Mean ± SD or n						
			Number (n)	Gender (M/F)	Age (years)	Diabetes mellitus duration (years)	Glycosylated hemoglobin (%)	The mean skin autofluorescence (Children with type1 diabetes)(AU)	The mean skin autofluorescence (Normal children)(AU)
Pascal, 2012 (17)	Case-control study	France	T1:52 Normal:28	T1:29/23 Normal:13/15	T1:12.00 ± 4.57* Normal:11.00 ± 4.69*	5.94 ± 2.51*	8.00 ± 0.84*	1.36 ± 0.32	1.20 ± 0.24
Josine, 2016 (18)	retrospective cohort study	The Netherlands	T1:77 Normal:118	T1:39/38 Normal:77/41	T1:15.30 ± 0.71* Normal:14.38 ± 0.45*	6.53 ± 4.53*	8.46 ± 1.02*	1.40 ± 0.06	1.14 ± 0.14
Y.H. Cho, 2014 (19)	Case-control study	Australia	T1:78 Normal:70	/	T1:17.40 ± 3.80 Normal:17.60 ± 6.00	10.10 ± 4.00	8.80 ± 1.60	1.43 ± 0.04	1.22 ± 0.04
H. Cho, 2016 (20)	Case-control study	Australia	T1:135 Normal:40	T1:69/66 Normal:22/18	T1:15.60 ± 2.10 Normal:15.40 ± 4.40	8.70 ± 3.50	8.70 ± 1.50	1.23 ± 0.27	1.14 ± 0.29

*For research reports partially or entirely in five-digit abstracts, data adjustments follow the guidelines outlined in the referenced literature (22–25).

### 3.2 Meta-analysis

#### 3.2.1 Relationship between SAF and other variables

**Figure 2:**
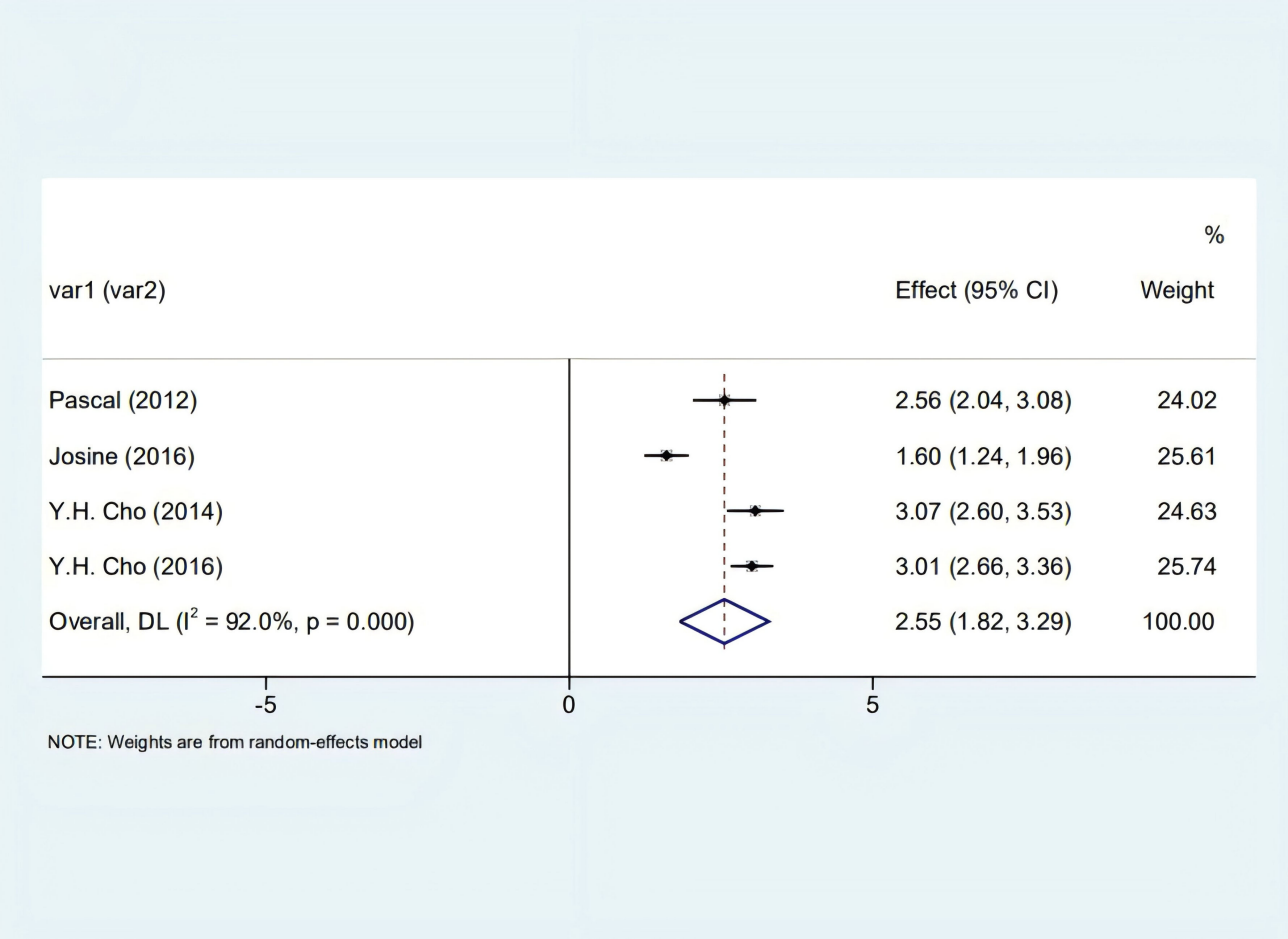
Forest plot illustrating SAF's correlation with diabetes progression in patients.

**Figure 3:**
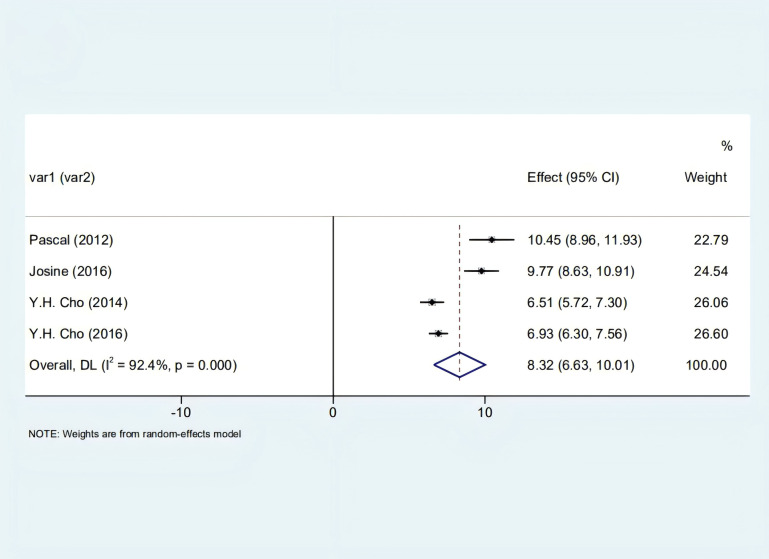
Forest plot depicting the correlation between SAF and glycosylated hemoglobin in diabetic patients.

Our analysis identified a significant correlation between skin autofluorescence (SAF) and diabetes duration [2.55 (1.82, 3.29), I^2^ = 92.00%, p < 0.05] shown in **Figure 2**, and a prominent relationship with HbA1c levels [8.32 (6.63, 10.01), I^2^ = 92.4%, p < 0.05], as depicted in **Figure 3**. However, these findings are tentative due to the limited number of studies, underscoring the need for more research.

#### 3.2.2 SAF value of T1D and healthy children

**Figure 4:**
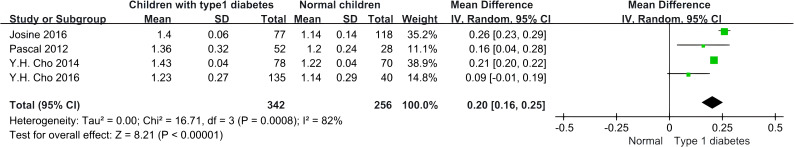
Forest plot displaying the comparison of SAF values between children with T1D and non-diabetic children.

Each study included in our analysis reported an association between skin autofluorescence (SAF) and children with Type 1 Diabetes (T1D). The data analysis showed significant heterogeneity among the four studies (I^2^ = 82.00%, p < 0.05). Using the random effects model, we found a positive correlation between higher SAF levels and T1D in children [0.20 (0.16, 0.25)], indicating that, in these studies, children with T1D exhibited higher SAF values compared to their healthy counterparts, as illustrated in **Figure 4**.

#### 3.2.3 Sensitivity analysis and publication bias

**Figure 5:**
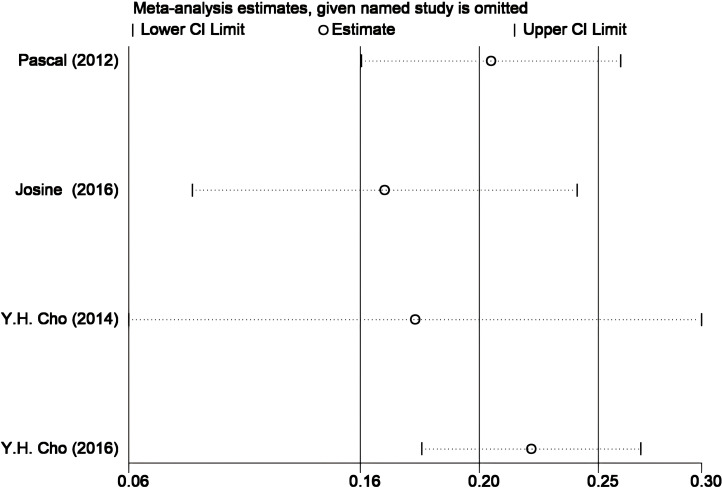
Outcomes from the STATA sensitivity analysis.

**Figure 6:**
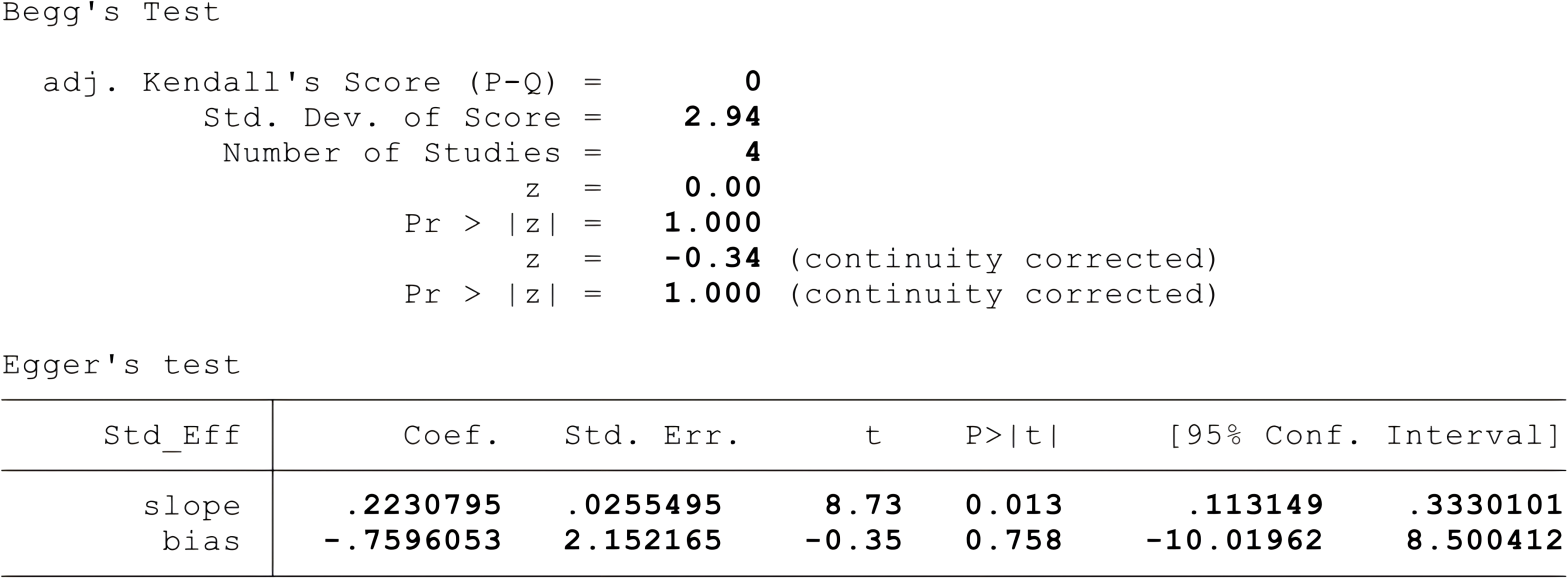
Outcomes of Egger's and Begg's tests across the four studies.

Given the small number and high heterogeneity of the included studies, we performed sensitivity and bias analyses. The STATA-based sensitivity analysis (**Figure 5**) showed no significant differences, and Egger’s and Begg’s tests indicated no notable bias in the four studies (p > 0.05). Nonetheless, the necessity for further research is underscored by the overlap in research teams and the small sample sizes in two studies (19, 20), as detailed in **Figure 6**.

## 4 Discussion

Our review suggests a positive correlation between elevated skin autofluorescence (SAF) and Type 1 Diabetes (T1D) in children, as well as a link between T1D progression and HbA1c levels. While SAF is not yet a direct diagnostic tool for T1D and its complications, it serves as a valuable clinical factor in assessing the overall status of the disease in children. The limited and heterogeneous nature of current research on T1D and SAF in children warrants cautious interpretation of results. Concurrently, we advocate for increased focus on this area to identify more suitable non-invasive diagnostic tools for children with T1D.

T1D, predominant among children and adolescents, considerably affects their healthy growth. Its complications are more severe and frequent compared to those in adult diabetes. Comprehending the pathology and complications of T1D in youth is crucial for prevention strategies (3–5). Traditionally, T1D diagnosis relied on fasting blood glucose levels, random glucose concentrations, oral glucose tolerance tests, or glycated hemoglobin. However, HbA1c, despite its long-term monitoring capability, is not ideal for T1D diagnosis due to rapid blood sugar fluctuations in patients (3). Consequently, new detection methods are necessary. Growing research highlights SAF’s effectiveness in diagnosing diabetes and its associated complications, including microvascular and macrovascular diseases and diabetic nephropathy, especially in adults (14–16). Our goal is to establish SAF as a non-invasive tool for T1D management, particularly for early assessment of complication risks, thus providing valuable clinical insights.

Josine C’s team (18) found a strong correlation between HbA1c and SAF, suggesting that high blood sugar may cause glycation damage to long-lived proteins. Cho (19, 20) noted that diabetic children exhibit higher skin fluorescence, significantly associated with potential retinopathy and cardiac autonomic neuropathy. This condition might be linked to age-induced protein cross-linking, leading to vascular stiffening, structural alterations, and functional changes in blood vessels. These intriguing findings warrant additional research.

Regarding kidney disease, Josephine M’s team (26) discovered a correlation between epidermal growth factor receptor (EGFR) and diabetic kidney disease (DKD) risks across all models. Increased EGFR in T1D patients is associated with prolonged diabetes duration and heightened DKD risk. SAF, cyclic age, and urine age do not independently predict DKD risks. Further information on the link between SAF and nephropathy in T1D children is lacking, precluding current discussion; however, based on cardiovascular disease theories, SAF may also be connected to renal vascular damage.

Mácsai’s study (27), employing desorption electrospray ionization mass spectrometry (DESI-MS) to investigate the link between SAF and certain skin tissue compounds, particularly intrigues us. Various reports imply a connection between these compounds and diabetes, along with its complications, prompting investigation into their biomarker potential for diabetes detection. This theory requires additional research for confirmation.

Research has shown that SAF values are influenced by various factors such as age, smoking history, physical activity, and renal function. Duda-sobczak A noted elevated SAF levels in women compared to men. Analyses in both adults and young adults without diabetes revealed no significant differences in SAF levels. Other studies, including MookKanamori et al. (28), have generally found higher SAF values in women, especially those with type 2 diabetes, and similar trends were observed in the Saudi population, though diabetes status was not always specified (29). However, most studies report no gender differences in SAF (30). Additional lifestyle elements like sun exposure and coffee intake might also affect SAF levels. As researchers specializing in dermatology, our team focuses particularly on skin organ tests related to SAF (31).

The core principle of skin autofluorescence (SAF) detection is based on the fluorescent properties of advanced glycation end products (AGEs). AGEs (such as pentosidine, crossline, etc.) emit fluorescence at 300–600 nm under 370 nm excitation, a characteristic utilized by the AGE Reader^®^ for noninvasive measurement (11). Although skin biopsy studies confirm a strong correlation between SAF and tissue AGEs levels (11–13), other skin components (e.g., collagen) may influence fluorescence signals through multiple mechanisms. First, collagen, as the primary deposition site for AGEs, has a long half-life (10–15 years), making it an ideal medium for reflecting long-term glycemic control (9). However, its cross-linking or degradation may indirectly alter SAF values (30). Second, structural proteins such as elastin and keratin, along with endogenous metabolites like flavins and lipofuscin, may contribute to background fluorescence (27). Additionally, exogenous factors such as ultraviolet exposure and smoking could modify skin composition via oxidative stress, further compromising signal specificity (10). These complex interactions collectively lead to heterogeneity in SAF measurements (e.g., meta-analysis I²=82%) (32), highlighting the need for multimodal techniques to disentangle the biological origins of fluorescence signals.

Despite fluorescence interference, the robust correlation between SAF and age can be explained as follows: (1) Age-dependent accumulation of AGEs​ is the dominant factor. Physiological AGEs formation increases with age, while metabolic dysregulation in diabetes accelerates this process (approximately 0.05 AU/year), resulting in signal intensities far exceeding other components (9, 12). (2) Collagen-AGEs co-evolution. Collagen content and cross-linking increase with age, and its intrinsic weak fluorescence (400–450 nm) partially overlaps with the AGEs spectral signature (420–600 nm), amplifying age-related signal enhancement (10, 30). (3) Technical optimization minimizes interference. The AGE Reader^®^ selectively employs 370 nm excitation and 420–600 nm detection windows to prioritize AGEs-specific peaks, while algorithms correct for variations in skin type and thickness (18, 27). Additionally, rapid hyperglycemia-driven AGEs generation in pediatric diabetes populations (e.g., type 1 diabetes) (26) and collinearity between age and metabolic factors in statistical models (16) further accentuate the apparent SAF-age association.

AGEs formation is directly regulated by blood glucose levels through the following mechanisms: (1) Non-enzymatic glycation cascade. Hyperglycemia accelerates the reaction of reducing sugars (e.g., glucose) with protein amino groups, forming Schiff bases and Amadori products, which are ultimately oxidized into stable AGEs (e.g., pentosidine, carboxymethyllysine [CML]) (6). (2) Imbalance between production and clearance. In diabetes, AGEs generation (glucose-dependent) outpaces clearance by macrophages and endothelial cells. Furthermore, AGEs-RAGE receptor interactions exacerbate inflammation and metabolic dysregulation via NF-κB signaling, creating a pathological feedback loop (32). (3) Tissue-specific accumulation. Long-lived proteins such as skin collagen (half-life: 10–15 years) enable progressive AGEs deposition, reflecting cumulative glycemic exposure over years rather than short-term HbA1c fluctuations (31). This mechanism not only explains the direct correlation between SAF and glycemia but also supports SAF as a tool for long-term diabetes monitoring. Future research should clarify subtype-specific contributions of AGEs (e.g., CML *vs*. pentosidine) to optimize clinical utility (6, 9).

## 5 Limitations

This review and analysis support the use of SAF as a clinical predictive tool for children’s diabetes and its complications. However, this review and analysis have several limitations that warrant mention. First, the included studies vary, encompassing case comparisons, prospective and retrospective analyses, but lack randomized controlled trials (RCTs) or more conclusive research. Second, the quantity of included studies is limited, with two conducted by the same lead author. Third, the high SAF values raise concerns about potential biases, as not all influencing factors were analyzed and discussed. Fourth, the significant heterogeneity of the study, primarily attributed to the small sample sizes, poses a challenge. Meanwhile, due to the scarcity of the latest research, more and newer discoveries will continue. Additionally, this study ventures into a relatively new research area. It’s challenging to ascertain if the actual findings align with those reported, due to potential research deviations.

## 6 Conclusions

This research advocates for employing SAF measurement as a non-invasive indicator for T1D and its complications in children, though further extensive studies and prolonged follow-up are necessary for concrete conclusions and detailed data regarding T1D complications. Considering the skin’s status as the body’s largest organ, its full potential remains largely untapped. We aim to simplify, refine, and enhance complex or invasive skin-related diagnoses and treatments through interdisciplinary collaboration.” This revised version eliminates redundancy, maintaining the original message’s essence while focusing on the future potential of SAF measurements and skin research.

## Data Availability

The original contributions presented in the study are included in the article/supplementary material, further inquiries can be directed to the corresponding author/s.
